# Supplementary material for: Muslim undergraduate biology students’ evolution acceptance in the United States
Source: PLoS One. 2021 Aug 11;16(8):e0255588. doi: 10.1371/journal.pone.0255588 (PMC8357111; doi:10.1371/journal.pone.0255588)
Supplement: S1 File — (DOCX) [file pone.0255588.s001.docx]

**Supplementary Material**

Table of contents

| **Item** |  | **Pages** |
| --- | --- | --- |
| Copy of survey questions analyzed |  | 2 – 6 |
| Regression tables for difference analyses |  | 7 – 12 |
| Regression tables for factors influencing evolution acceptance  among different religious groups |  | 13 – 32 |
| Syntax for all analyses |  | 33 – 43 |

**Survey questions analyzed**

***Understanding of Evolution***

This portion of the survey is meant to determine how much you understand about current evolutionary theory as proposed by scientists. Please answer the following questions based on your understanding of evolution.

Please choose whether each statement is true, false, or you don’t know enough to answer based on your **understanding of evolution:**

1. Individuals don't evolve, species do.
2. Evolution is a progression towards more advanced species.
3. Mutations occur all the time.
4. Species evolve to be perfectly adapted to their environments.
5. In most groups of organisms, more offspring are born than survive.
6. Mutations can be passed down to the next generation.
7. More genetic variability makes a population more resistant to extinction.
8. Natural selection is the same thing as evolution.
9. The characteristics an organism acquires during their lifetime are often genetically passed down to their offspring.
10. Natural selection is the only cause of evolution.
11. The more recently species share a common ancestor, the more closely related they are.
12. Evolution means progression towards perfection.
13. Natural selection is a random process.
14. Natural selection means that only the smartest and physically strongest organisms survive.

***Interest in Evolution***

Please rate the following on a scale from 0 (not at all) to 10 (very much):

1. If you could, to what extent would you be interested in taking an elective course on evolution in the future?
2. If you could, to what extent would you be interested in doing research on evolution as an undergraduate?
3. To what extent can you see yourself studying evolution as part of your career?
4. To what extent can you see yourself becoming an evolutionary biologist?

***Acceptance of Common Ancestry***

Please indicate which of the following statements most closely represents your personal view, ***based on your personal opinion:***

1. All forms of life were first brought into being in their present form by God 6,000-10,000 years ago at the same time.
2. All forms of life were first brought into being in their present form by God at different times over billions of years.
3. Some forms of life evolved from earlier forms, but God created groups of organisms such as reptiles, birds, mammals, and humans separate from one another and organisms that currently exist have evolved slowly from those first creations.
4. Almost all forms of life evolved from earlier forms, but humans were created by God in their present form separate from the rest of life.
5. All forms of life evolved from earlier forms, but God intervenes from time to time to shape or override evolution.
6. All forms of life evolved from earlier forms, but God set up evolution from the start in a perfect way so that it would fulfill God’s purpose and no subsequent intervention was necessary.
7. All forms of life evolved from earlier forms, but life and evolution were first set in motion by God without a specific purpose or plan.
8. All forms of life evolved from earlier forms, but I'm not sure whether any God was involved in evolution.
9. All forms of life evolved from earlier forms, but no God has ever played a role in evolution.

***Acceptance of Human Evolution***

Please indicate whether you agree or disagree with the following statements, **based on your personal opinion.** (5-pt Likert-scale)

1. I think there is reliable evidence to support the theory that describes how humans were derived from ancestral primates.
2. I think that humans adapt, but they have not/do not evolve.
3. I think that the physical structures of humans are too complex to have evolved.
4. I think that humans and apes share an ancient ancestor.
5. I think that humans evolve.
6. I think that humans do not evolve; they can only change their behavior.
7. I think the many characteristics that humans share with other primates (i.e., chimpanzees, gorillas) can be best explained by our sharing a common ancestor.
8. I think physical variations in humans (i.e., eye color, skin color) were derived from the same processes that produce variation in other groups of organisms.

***Acceptance of Macroevolution***

Please indicate whether you agree or disagree with the following statements, **based on your personal opinion.** (5-pt Likert-scale)

1. I think that new species evolved from ancestral species.
2. I think that the fossil evidence that scientists use to support evolutionary theory is weak and inconclusive.
3. I think there are a large number of fossils found all around the world that support the ideas that organisms evolve into new species over time.
4. I think all complex organisms evolved from single-celled organisms.
5. I think that new species evolve from a lot of small changes occurring over relatively long periods of time.
6. I think there is little or no observable evidence to support the theory that describes how one species of organism evolves from a different ancestral form.
7. I think the forms and diversity of organisms have changed dramatically over time.
8. I think that all organisms come from a single common ancestor.

***Acceptance of Microevolution***

Please indicate whether you agree or disagree with the following statements, **based on your personal opinion.** (5-pt Likert-scale)

1. I think that organisms, as they exist now, are perfectly adapted to their natural environments and so will not continue to change.
2. I think all groups of organisms will continue to change.
3. I think there are a large number of examples of organisms that have undergone evolutionary changes within the species (i.e., antibiotic resistance in bacteria, production of new strains of the flu virus).
4. I think that species were created to be perfectly suited to their environment, so they do not change.
5. I don't accept the idea that a species of organism will evolve new traits over time.
6. I think there is an abundance of observable evidence to support the theory describing how variations within a species can happen.
7. I think that species exist today in exactly the same shape and form in which they always have.
8. I think there is overwhelming evidence supporting the theory of evolution to explain how variations in a species develop over time.

***Religious Affiliation***

I most closely identify as:

- Buddhist
- Christian
- Hindu
- Jewish
- Muslim
- I don't identify with a religion
- Option not available, please describe _________________________
- Prefer not to answer

If “Christian” is chosen:

With what denomination of Christianity do you most closely identify?

- Catholic
- Jehovah's Witness
- Orthodox
- Nondenominational
- Protestant
- The Church of Jesus Christ of Latter-day Saints
- Option not available, please describe _________________________
- Prefer not to answer

If “I don’t identify with a religion” is chosen:

- I most closely identify as:
- Atheist (believes that God does not exist)
- Agnostic (does not have a definite belief about whether God exists or not)
- Option not available, please describe: __________________________
- Prefer not to answer

***Religiosity***

Please indicate how much you agree or disagree with the following statements: (5-pt Likert-scale)

1. I attend religious services regularly (when they are available)
2. I believe in God
3. I consider myself a religious person
4. I consider myself a spiritual person

***Major***

Is your major in biology? (includes biomedical sciences, biology and society, conservation biology, genetics, neurobiology/physiology/behavior, microbiology, medical microbiology, molecular bioscience, neuroscience)

- Yes
- No
- I'm not sure (please describe): _______________________________

***Gender***

I most closely identify as:

- Woman
- Man
- Nonbinary
- Decline to state
- Please describe your gender identity if the best option is not listed: __________

***Race/ethnicity:***

What is your ethnicity? Please select all that apply.

- Asian (East Asian, Southeast Asian, South Asian, West Asian, Middle Eastern)
- Black
- Latinx
- Native American, American Indian, or Alaskan Native
- Native Hawaiian or Other Pacific Islander
- White
- Decline to state
- Option not available, please describe: ______________________________

***International Status:***

Were you born in the United States?

- Yes
- No
- Decline to state

**Regression tables looking at differences between religious group acceptance of evolution.**

Table S1: Summary of linear regression results using evolution understanding as the dependent variable, *R*^2^ = .07, *F* (17, 7891) = 36.24, *p* < .001. Variables that are significant at the .05 level are bolded. The reference group for gender is “Man,” race/ethnicity “White,” and religious affiliation “Muslim.”

|  | **Unstandardized Coefficients** | | **Standardized Coefficients** |  | | **95% Confidence Intervals for B** | |
| --- | --- | --- | --- | --- | --- | --- | --- |
|  | **B** | **Std. Error** | **Beta (β)** | **t** | **Significance**  **(p-value)** | **Lower Bound** | **Upper Bound** |
| (Constant) | .665 | .013 |  | 50.775 | .000 | .639 | .690 |
| **Biology Major** | .035 | .004 | .106 | 9.653 | .000 | .028 | .042 |
| Woman | -.004 | .004 | -.012 | -1.126 | .260 | -.012 | .003 |
| **Nonbinary Gender** | .059 | .024 | .027 | 2.506 | .012 | .013 | .106 |
| **Asian** | -.022 | .006 | -.049 | -3.587 | .000 | -.034 | -.010 |
| **Black** | -.086 | .008 | -.122 | -10.694 | .000 | -.102 | -.070 |
| **Latinx** | -.080 | .005 | -.178 | -14.816 | .000 | -.091 | -.069 |
| **Multiracial** | -.014 | .006 | -.026 | -2.315 | .021 | -.026 | -.002 |
| **Other race** | -.045 | .018 | -.028 | -2.549 | .011 | -.079 | -.010 |
| **Born in USA** | .024 | .006 | .047 | 3.927 | .000 | .012 | .037 |
| Protestant | .013 | .012 | .034 | 1.124 | .261 | -.010 | .036 |
| CJCLDS | .020 | .013 | .036 | 1.576 | .115 | -.005 | .045 |
| Catholic | -.002 | .012 | -.006 | -.185 | .853 | -.025 | .021 |
| Jewish | .012 | .017 | .010 | .717 | .473 | -.021 | .046 |
| Hindu | .001 | .017 | .001 | .036 | .971 | -.032 | .033 |
| Buddhist | .016 | .016 | .014 | .959 | .337 | -.016 | .047 |
| **Agnostic** | .034 | .012 | .089 | 2.927 | .003 | .011 | .057 |
| **Atheist** | .067 | .013 | .108 | 5.268 | .000 | .042 | .092 |

Table S2: Summary of linear regression results using interest in evolution as the dependent variable, *R*^2^ = .17, *F* (17, 6501) = 82.45, *p* < .001. Variables that are significant at the .05 level are bolded. The reference group for gender is “Man,” race/ethnicity “White,” and religious affiliation “Muslim.”

|  | **Unstandardized Coefficients** | | **Standardized Coefficients** |  | | **95% Confidence Intervals for B** | |
| --- | --- | --- | --- | --- | --- | --- | --- |
|  | **B** | **Std. Error** | **Beta (β)** | **t** | **Significance**  **(p-value)** | **Lower Bound** | **Upper Bound** |
| (Constant) | 3.321 | .197 |  | 16.843 | .000 | 2.934 | 3.707 |
| **Biology Major** | 1.279 | .056 | .261 | 22.869 | .000 | 1.169 | 1.388 |
| **Woman** | -.246 | .060 | -.047 | -4.089 | .000 | -.364 | -.128 |
| Nonbinary Gender | .464 | .348 | .015 | 1.331 | .183 | -.219 | 1.146 |
| Asian | -.019 | .096 | -.003 | -.200 | .842 | -.208 | .169 |
| Black | -.060 | .122 | -.006 | -.492 | .622 | -.298 | .178 |
| **Latinx** | .326 | .081 | .051 | 4.030 | .000 | .167 | .484 |
| Multiracial | .170 | .094 | .021 | 1.811 | .070 | -.014 | .354 |
| Other race | .162 | .288 | .006 | .562 | .574 | -.403 | .727 |
| **Born in USA** | -.377 | .095 | -.049 | -3.967 | .000 | -.563 | -.191 |
| **Protestant** | -.803 | .175 | -.143 | -4.591 | .000 | -1.147 | -.460 |
| **CJCLDS** | -.692 | .193 | -.079 | -3.578 | .000 | -1.071 | -.313 |
| Catholic | .169 | .176 | .030 | .961 | .337 | -.176 | .514 |
| **Jewish** | .777 | .262 | .043 | 2.960 | .003 | .262 | 1.291 |
| Hindu | .438 | .251 | .026 | 1.745 | .081 | -.054 | .930 |
| **Buddhist** | 1.355 | .252 | .078 | 5.370 | .000 | .860 | 1.850 |
| **Agnostic** | .866 | .174 | .153 | 4.992 | .000 | .526 | 1.206 |
| **Atheist** | 1.133 | .191 | .123 | 5.926 | .000 | .758 | 1.508 |

Table S3: Summary of binary logistic regression results using acceptance of common ancestry of life on Earth as the dependent variable, *Cox & Snell R^2^* = .32, (χ^2^ (17) = 3045.11, p < .001)

|  |  | |  |  | |  | **95% Confidence Intervals for B** | |
| --- | --- | --- | --- | --- | --- | --- | --- | --- |
|  | **B** | **Std. Error** | **Wald** | **df** | **Significance**  **(p-value)** | **Exp(B)** | **Lower Bound** | **Upper Bound** |
| (Constant) | -.169 | .186 | .827 | 1 | .363 | .845 |  |  |
| **Biology Major** | .119 | .057 | 4.321 | 1 | .038 | 1.126 | 1.007 | 1.260 |
| **Woman** | -.365 | .062 | 35.174 | 1 | .000 | .694 | .615 | .783 |
| Nonbinary Gender | -.935 | .497 | 3.538 | 1 | .060 | .392 | .148 | 1.040 |
| Asian | -.135 | .103 | 1.712 | 1 | .191 | .874 | .714 | 1.070 |
| **Black** | -.674 | .124 | 29.533 | 1 | .000 | .510 | .400 | .650 |
| **Latinx** | -.301 | .082 | 13.396 | 1 | .000 | .740 | .630 | .870 |
| Multiracial | .075 | .096 | .612 | 1 | .434 | 1.078 | .893 | 1.303 |
| Other race | .096 | .274 | .124 | 1 | .725 | 1.101 | .644 | 1.883 |
| Born in USA | -.139 | .102 | 1.829 | 1 | .176 | .871 | .712 | 1.064 |
| Protestant | -.072 | .158 | .207 | 1 | .649 | .930 | .682 | 1.269 |
| **CJCLDS** | -.383 | .173 | 4.928 | 1 | .026 | .682 | .486 | .956 |
| **Catholic** | .672 | .158 | 18.045 | 1 | .000 | 1.958 | 1.436 | 2.669 |
| **Jewish** | 1.800 | .249 | 52.249 | 1 | .000 | 6.052 | 3.714 | 9.860 |
| **Hindu** | 1.834 | .240 | 58.470 | 1 | .000 | 6.257 | 3.911 | 10.012 |
| **Buddhist** | 2.678 | .283 | 89.492 | 1 | .000 | 14.558 | 8.358 | 25.356 |
| **Agnostic** | 3.485 | .179 | 377.320 | 1 | .000 | 32.624 | 22.952 | 46.372 |
| **Atheist** | 6.243 | .724 | 74.450 | 1 | .000 | 514.628 | 124.616 | 2125.267 |

Table S4: Summary of linear regression results using human evolution acceptance as the dependent variable, *R*^2^ = .25, *F* (17, 7891) = 150.88, *p* < .001. Variables that are significant at the .05 level are bolded. The reference group for gender is “Man,” race/ethnicity “White,” and religious affiliation “Muslim.”

|  | **Unstandardized Coefficients** | | **Standardized Coefficients** |  | | **95% Confidence Intervals for B** | |
| --- | --- | --- | --- | --- | --- | --- | --- |
|  | **B** | **Std. Error** | **Beta (β)** | **t** | **Significance**  **(p-value)** | **Lower Bound** | **Upper Bound** |
| (Constant) | 3.368 | .059 |  | 57.184 | .000 | 3.252 | 3.483 |
| **Biology Major** | .088 | .016 | .053 | 5.360 | .000 | .056 | .120 |
| **Woman** | -.071 | .018 | -.041 | -4.063 | .000 | -.105 | -.037 |
| Nonbinary Gender | .073 | .107 | .007 | .685 | .493 | -.136 | .283 |
| **Asian** | -.112 | .027 | -.051 | -4.078 | .000 | -.165 | -.058 |
| **Black** | -.093 | .036 | -.026 | -2.571 | .010 | -.164 | -.022 |
| **Latinx** | -.123 | .024 | -.055 | -5.077 | .000 | -.171 | -.076 |
| Multiracial | .028 | .028 | .011 | 1.035 | .301 | -.025 | .082 |
| Other race | -.137 | .079 | -.017 | -1.735 | .083 | -.292 | .018 |
| **Born in USA** | .070 | .028 | .027 | 2.512 | .012 | .015 | .125 |
| **Protestant** | -.150 | .053 | -.079 | -2.859 | .004 | -.254 | -.047 |
| **CJCLDS** | -.258 | .057 | -.094 | -4.542 | .000 | -.370 | -.147 |
| **Catholic** | .368 | .053 | .190 | 6.952 | .000 | .264 | .472 |
| **Jewish** | .655 | .077 | .110 | 8.501 | .000 | .504 | .806 |
| **Hindu** | .619 | .075 | .107 | 8.296 | .000 | .472 | .765 |
| **Buddhist** | .707 | .073 | .126 | 9.678 | .000 | .564 | .850 |
| **Agnostic** | .704 | .052 | .371 | 13.516 | .000 | .602 | .806 |
| **Atheist** | .930 | .057 | .301 | 16.225 | .000 | .818 | 1.042 |

Table S5: Summary of linear regression results using macroevolution acceptance as the dependent variable, *R*^2^ = .18, *F* (17, 7891) = 104.50, *p* < .001. Variables that are significant at the .05 level are bolded. The reference group for gender is “Man,” race/ethnicity “White,” and religious affiliation “Muslim.”

|  | **Unstandardized Coefficients** | | **Standardized Coefficients** |  | | **95% Confidence Intervals for B** | |
| --- | --- | --- | --- | --- | --- | --- | --- |
|  | **B** | **Std. Error** | **Beta (β)** | **t** | **Significance**  **(p-value)** | **Lower Bound** | **Upper Bound** |
| (Constant) | 3.539 | .050 |  | 71.477 | .000 | 3.442 | 3.636 |
| **Biology Major** | .097 | .014 | .073 | 7.046 | .000 | .070 | .124 |
| **Woman** | -.051 | .015 | -.036 | -3.490 | .000 | -.080 | -.023 |
| Nonbinary Gender | .053 | .090 | .006 | .585 | .558 | -.123 | .229 |
| Asian | -.046 | .023 | -.026 | -2.001 | .045 | -.091 | -.001 |
| Black | -.038 | .030 | -.013 | -1.234 | .217 | -.097 | .022 |
| Latinx | -.040 | .020 | -.022 | -1.966 | .049 | -.080 | .000 |
| Multiracial | .045 | .023 | .021 | 1.924 | .054 | -.001 | .090 |
| Other race | -.039 | .066 | -.006 | -.584 | .559 | -.169 | .091 |
| **Born in USA** | .088 | .023 | .041 | 3.728 | .000 | .042 | .134 |
| **Protestant** | -.152 | .044 | -.098 | -3.427 | .001 | -.238 | -.065 |
| **CJCLDS** | -.159 | .048 | -.071 | -3.316 | .001 | -.252 | -.065 |
| **Catholic** | .193 | .045 | .123 | 4.335 | .000 | .106 | .280 |
| **Jewish** | .364 | .065 | .075 | 5.621 | .000 | .237 | .491 |
| **Hindu** | .375 | .063 | .081 | 5.982 | .000 | .252 | .498 |
| **Buddhist** | .417 | .061 | .092 | 6.783 | .000 | .296 | .537 |
| **Agnostic** | .444 | .044 | .289 | 10.128 | .000 | .358 | .530 |
| **Atheist** | .669 | .048 | .268 | 13.880 | .000 | .574 | .763 |

Table S6: Summary of linear regression results using microevolution acceptance as the dependent variable, *R*^2^ = .08, *F* (17, 7891) = 42.53, *p* < .001. Variables that are significant at the .05 level are bolded. The reference group for gender is “Man,” race/ethnicity “White,” and religious affiliation “Muslim.”

|  | **Unstandardized Coefficients** | | **Standardized Coefficients** |  | | **95% Confidence Intervals for B** | |
| --- | --- | --- | --- | --- | --- | --- | --- |
|  | **B** | **Std. Error** | **Beta (β)** | **t** | **Significance**  **(p-value)** | **Lower Bound** | **Upper Bound** |
| (Constant) | 4.028 | .043 |  | 92.645 | .000 | 3.943 | 4.113 |
| **Biology Major** | .064 | .012 | .058 | 5.314 | .000 | .040 | .088 |
| **Woman** | .032 | .013 | .027 | 2.468 | .014 | .007 | .057 |
| **Nonbinary Gender** | .160 | .079 | .022 | 2.024 | .043 | .005 | .314 |
| **Asian** | -.110 | .020 | -.075 | -5.460 | .000 | -.150 | -.071 |
| **Black** | -.159 | .027 | -.068 | -5.961 | .000 | -.212 | -.107 |
| **Latinx** | -.121 | .018 | -.081 | -6.755 | .000 | -.156 | -.086 |
| Multiracial | .009 | .020 | .005 | .426 | .670 | -.031 | .048 |
| **Other race** | -.154 | .058 | -.029 | -2.635 | .008 | -.268 | -.039 |
| **Born in USA** | .101 | .021 | .057 | 4.876 | .000 | .060 | .141 |
| Protestant | .005 | .039 | .004 | .141 | .888 | -.071 | .082 |
| CJCLDS | .058 | .042 | .031 | 1.386 | .166 | -.024 | .140 |
| **Catholic** | .122 | .039 | .094 | 3.122 | .002 | .045 | .199 |
| **Jewish** | .174 | .057 | .043 | 3.058 | .002 | .062 | .285 |
| **Hindu** | .195 | .055 | .051 | 3.547 | .000 | .087 | .303 |
| **Buddhist** | .284 | .054 | .075 | 5.275 | .000 | .179 | .390 |
| **Agnostic** | .308 | .038 | .242 | 8.015 | .000 | .233 | .384 |
| **Atheist** | .424 | .042 | .205 | 10.022 | .000 | .341 | .507 |

**MUSLIM STUDENT ONLY REGRESSIONS**

Table S7: Summary of binary logistic regression results among Muslim students only using acceptance of the common ancestry of life on Earth as the dependent variable, *Cox & Snell R^2^* = .10, (χ^2^ (5) = 21.847, p < .001)

|  |  | |  |  | |  | **95% Confidence Intervals for B** | |
| --- | --- | --- | --- | --- | --- | --- | --- | --- |
|  | **B** | **Std. Error** | **Wald** | **df** | **Significance**  **(p-value)** | **Exp(B)** | **Lower Bound** | **Upper Bound** |
| (Constant) | 2.356 | .969 | 5.913 | 1 | .015 | 10.545 |  |  |
| Evolution Understanding | .954 | .938 | 1.033 | 1 | .310 | 2.595 | .412 | 16.327 |
| **Religiosity** | -.888 | .220 | 16.334 | 1 | .000 | .412 | .268 | .633 |
| Biology Major | .128 | .313 | .167 | 1 | .683 | 1.136 | .616 | 2.097 |
| **Woman** | -.653 | .301 | 4.703 | 1 | .030 | .521 | .289 | .939 |
| Born in the U.S. | .188 | .339 | .308 | 1 | .579 | 1.207 | .621 | 2.347 |

Table S8: Summary of linear regression results among Muslim students only using human evolution acceptance as the dependent variable, *R*^2^ = .17, *F* (5, 213) = 10.13, *p* < .001. Variables that are significant at the .05 level are bolded. The reference group for gender is “Man” and race/ethnicity “White.”

|  | **Unstandardized Coefficients** | | **Standardized Coefficients** |  | | **95% Confidence Intervals for B** | |
| --- | --- | --- | --- | --- | --- | --- | --- |
|  | **B** | **Std. Error** | **Beta (β)** | **t** | **Significance**  **(p-value)** | **Lower Bound** | **Upper Bound** |
| (Constant) | 4.398 | .325 |  | 13.520 | .000 | 3.757 | 5.040 |
| **Evolution Understanding** | .983 | .319 | .199 | 3.078 | .002 | .353 | 1.612 |
| **Religiosity** | -.468 | .071 | -.423 | -6.625 | .000 | -.608 | -.329 |
| Biology Major | .026 | .105 | .015 | .245 | .806 | -.180 | .232 |
| Woman | -.096 | .101 | -.059 | -.951 | .343 | -.295 | .103 |
| Born in the U.S. | .211 | .113 | .121 | 1.868 | .063 | -.012 | .434 |

Table S9: Summary of linear regression results among Muslim students only using macroevolution acceptance as the dependent variable, *R*^2^ = .09, *F* (5, 213) = 5.35, *p* < .001. Variables that are significant at the .05 level are bolded. The reference group for gender is “Man” and race/ethnicity “White.”

|  | **Unstandardized Coefficients** | | **Standardized Coefficients** |  | | **95% Confidence Intervals for B** | |
| --- | --- | --- | --- | --- | --- | --- | --- |
|  | **B** | **Std. Error** | **Beta (β)** | **t** | **Significance**  **(p-value)** | **Lower Bound** | **Upper Bound** |
| (Constant) | 3.588 | .260 |  | 13.810 | .000 | 3.076 | 4.101 |
| **Evolution Understanding** | 1.003 | .255 | .267 | 3.933 | .000 | .500 | 1.505 |
| **Religiosity** | -.188 | .056 | -.223 | -3.328 | .001 | -.299 | -.077 |
| Biology Major | -.005 | .084 | -.004 | -.065 | .948 | -.170 | .159 |
| Woman | -.041 | .081 | -.033 | -.505 | .614 | -.200 | .118 |
| Born in the U.S. | .137 | .090 | .102 | 1.514 | .132 | -.041 | .315 |

Table S10: Summary of linear regression results among Muslim students only using microevolution acceptance as the dependent variable, *R*^2^ = .10, *F* (5, 213) = 5.80, *p* < .001. Variables that are significant at the .05 level are bolded. The reference group for gender is “Man” and race/ethnicity “White.”

|  | **Unstandardized Coefficients** | | **Standardized Coefficients** |  | | **95% Confidence Intervals for B** | |
| --- | --- | --- | --- | --- | --- | --- | --- |
|  | **B** | **Std. Error** | **Beta (β)** | **t** | **Significance**  **(p-value)** | **Lower Bound** | **Upper Bound** |
| (Constant) | 3.895 | .252 |  | 15.488 | .000 | 3.400 | 4.391 |
| **Evolution Understanding** | 1.097 | .247 | .300 | 4.447 | .000 | .611 | 1.584 |
| **Religiosity** | -.166 | .055 | -.202 | -3.032 | .003 | -.273 | -.058 |
| Biology Major | -.021 | .081 | -.017 | -.259 | .796 | -.180 | .138 |
| Woman | .043 | .078 | .035 | .550 | .583 | -.111 | .197 |
| Born in the U.S. | .093 | .087 | .072 | 1.065 | .288 | -.079 | .265 |

**PROTESTANT STUDENT ONLY REGRESSSIONS**

Table S11: Summary of binary logistic regression results among Protestant students only using acceptance of the common ancestry of life on Earth as the dependent variable, *Cox & Snell R^2^* = .10, (χ^2^ (5) = 200.846, p < .001)

|  |  | |  |  | |  | **95% Confidence Intervals for B** | |
| --- | --- | --- | --- | --- | --- | --- | --- | --- |
|  | **B** | **Std. Error** | **Wald** | **df** | **Significance**  **(p-value)** | **Exp(B)** | **Lower Bound** | **Upper Bound** |
| (Constant) | 1.425 | .404 | 12.448 | 1 | .000 | 4.159 |  |  |
| **Evolution Understanding** | 1.616 | .323 | 25.098 | 1 | .000 | 5.032 | 2.674 | 9.468 |
| **Religiosity** | -.832 | .069 | 145.897 | 1 | .000 | .435 | .380 | .498 |
| **Biology Major** | .241 | .103 | 5.512 | 1 | .019 | 1.272 | 1.041 | 1.555 |
| **Woman** | -.269 | .115 | 5.511 | 1 | .019 | .764 | .610 | .957 |
| Born in the U.S. | .140 | .222 | .399 | 1 | .527 | 1.150 | .745 | 1.777 |

Table S12: Summary of linear regression results among Protestant students only using human evolution acceptance as the dependent variable, *R*^2^ = .15, *F* (5, 1946) = 71.531, *p* < .001. Variables that are significant at the .05 level are bolded. The reference group for gender is “Man” and race/ethnicity “White.”

|  | **Unstandardized Coefficients** | | **Standardized Coefficients** |  | | **95% Confidence Intervals for B** | |
| --- | --- | --- | --- | --- | --- | --- | --- |
|  | **B** | **Std. Error** | **Beta (β)** | **t** | **Significance**  **(p-value)** | **Lower Bound** | **Upper Bound** |
| (Constant) | 4.175 | .146 |  | 28.525 | .000 | 3.888 | 4.462 |
| **Evolution Understanding** | .913 | .115 | .167 | 7.969 | .000 | .689 | 1.138 |
| **Religiosity** | -.412 | .024 | -.355 | -16.997 | .000 | -.459 | -.364 |
| **Biology Major** | .079 | .037 | .044 | 2.116 | .035 | .006 | .153 |
| Woman | -.031 | .043 | -.015 | -.727 | .467 | -.115 | .053 |
| Born in the U.S. | .088 | .078 | .024 | 1.139 | .255 | -.064 | .240 |

Table S13: Summary of linear regression results among Protestant students only using macroevolution acceptance as the dependent variable, *R*^2^ = .10, *F* (5, 1946) = 42.566, *p* < .001. Variables that are significant at the .05 level are bolded. The reference group for gender is “Man” and race/ethnicity “White.”

|  | **Unstandardized Coefficients** | | **Standardized Coefficients** |  | | **95% Confidence Intervals for B** | |
| --- | --- | --- | --- | --- | --- | --- | --- |
|  | **B** | **Std. Error** | **Beta (β)** | **t** | **Significance**  **(p-value)** | **Lower Bound** | **Upper Bound** |
| (Constant) | 4.083 | .126 |  | 32.500 | .000 | 3.837 | 4.330 |
| **Evolution Understanding** | .544 | .098 | .120 | 5.529 | .000 | .351 | .737 |
| **Religiosity** | -.269 | .021 | -.279 | -12.923 | .000 | -.310 | -.228 |
| **Biology Major** | .119 | .032 | .080 | 3.715 | .000 | .056 | .183 |
| Woman | .029 | .037 | .017 | .800 | .424 | -.043 | .102 |
| Born in the U.S. | .017 | .067 | .005 | .255 | .799 | -.114 | .147 |

Table S14: Summary of linear regression results among Protestant students only using microevolution acceptance as the dependent variable, *R*^2^ = .12, *F* (5, 1946) = 52.538, *p* < .001. Variables that are significant at the .05 level are bolded. The reference group for gender is “Man” and race/ethnicity “White.”

|  | **Unstandardized Coefficients** | | **Standardized Coefficients** |  | | **95% Confidence Intervals for B** | |
| --- | --- | --- | --- | --- | --- | --- | --- |
|  | **B** | **Std. Error** | **Beta (β)** | **t** | **Significance**  **(p-value)** | **Lower Bound** | **Upper Bound** |
| (Constant) | 3.678 | .100 |  | 36.873 | .000 | 3.482 | 3.874 |
| **Evolution Understanding** | 1.075 | .078 | .295 | 13.764 | .000 | .922 | 1.228 |
| **Religiosity** | -.123 | .017 | -.159 | -7.433 | .000 | -.155 | -.090 |
| Biology Major | .038 | .026 | .032 | 1.480 | .139 | -.012 | .088 |
| **Woman** | .080 | .029 | .059 | 2.749 | .006 | .023 | .138 |
| **Born in the U.S.** | .145 | .053 | .059 | 2.753 | .006 | .042 | .249 |

**CHURCH OF JESUS CHRIST OF LATTER-DAY SAINTS STUDENT ONLY REGRESSIONS**

Table S15: Summary of binary logistic regression results among CJCLDS students only using acceptance of the common ancestry of life on Earth as the dependent variable, *Cox & Snell R^2^* = .03, (χ^2^ (5) = 20.742, p < .001)

|  |  | |  |  | |  | **95% Confidence Intervals for B** | |
| --- | --- | --- | --- | --- | --- | --- | --- | --- |
|  | **B** | **Std. Error** | **Wald** | **df** | **Significance**  **(p-value)** | **Exp(B)** | **Lower Bound** | **Upper Bound** |
| (Constant) | -.169 | .628 | .072 | 1 | .788 | .844 |  |  |
| **Evolution Understanding** | 1.678 | .532 | 9.948 | 1 | .002 | 5.352 | 1.887 | 15.180 |
| **Religiosity** | -.221 | .100 | 4.924 | 1 | .026 | .802 | .660 | .975 |
| Biology Major | .114 | .168 | .462 | 1 | .497 | 1.121 | .806 | 1.558 |
| Woman | .083 | .160 | .266 | 1 | .606 | 1.086 | .793 | 1.487 |
| **Born in the U.S.** | -1.001 | .359 | 7.778 | 1 | .005 | .367 | .182 | .743 |

Table S16: Summary of linear regression results among CJCLDS students only using human evolution acceptance as the dependent variable, *R*^2^ = .06, *F* (5, 774) = 11.008, *p* < .001. Variables that are significant at the .05 level are bolded. The reference group for gender is “Man” and race/ethnicity “White.”

|  | **Unstandardized Coefficients** | | **Standardized Coefficients** |  | | **95% Confidence Intervals for B** | |
| --- | --- | --- | --- | --- | --- | --- | --- |
|  | **B** | **Std. Error** | **Beta (β)** | **t** | **Significance**  **(p-value)** | **Lower Bound** | **Upper Bound** |
| (Constant) | 3.341 | .251 |  | 13.334 | .000 | 2.849 | 3.833 |
| **Evolution Understanding** | 1.112 | .193 | .205 | 5.761 | .000 | .733 | 1.491 |
| **Religiosity** | -.165 | .040 | -.147 | -4.154 | .000 | -.243 | -.087 |
| **Biology Major** | .129 | .065 | .070 | 1.987 | .047 | .002 | .256 |
| Woman | .022 | .061 | .013 | .363 | .717 | -.098 | .142 |
| Born in the U.S. | -.279 | .147 | -.066 | -1.899 | .058 | -.567 | .009 |

Table S17: Summary of linear regression results among CJCLDS students only using macroevolution acceptance as the dependent variable, *R*^2^ = .03, *F* (5, 774) = 6.54, *p* < .001. Variables that are significant at the .05 level are bolded. The reference group for gender is “Man” and race/ethnicity “White.”

|  | **Unstandardized Coefficients** | | **Standardized Coefficients** |  | | **95% Confidence Intervals for B** | |
| --- | --- | --- | --- | --- | --- | --- | --- |
|  | **B** | **Std. Error** | **Beta (β)** | **t** | **Significance**  **(p-value)** | **Lower Bound** | **Upper Bound** |
| (Constant) | 3.344 | .202 |  | 16.531 | .000 | 2.947 | 3.741 |
| **Evolution Understanding** | .776 | .156 | .180 | 4.976 | .000 | .470 | 1.082 |
| Religiosity | -.043 | .032 | -.048 | -1.335 | .182 | -.106 | .020 |
| Biology Major | .077 | .052 | .053 | 1.468 | .142 | -.026 | .180 |
| Woman | .012 | .049 | .009 | .253 | .800 | -.084 | .109 |
| **Born in the U.S.** | -.271 | .119 | -.081 | -2.289 | .022 | -.504 | -.039 |

Table S18: Summary of linear regression results among CJCLDS students only using microevolution acceptance as the dependent variable, *R*^2^ = .10, *F* (5, 774) = 17.35, *p* < .001. Variables that are significant at the .05 level are bolded. The reference group for gender is “Man” and race/ethnicity “White.”

|  | **Unstandardized Coefficients** | | **Standardized Coefficients** |  | | **95% Confidence Intervals for B** | |
| --- | --- | --- | --- | --- | --- | --- | --- |
|  | **B** | **Std. Error** | **Beta (β)** | **t** | **Significance**  **(p-value)** | **Lower Bound** | **Upper Bound** |
| (Constant) | 3.334 | .153 |  | 21.747 | .000 | 3.033 | 3.634 |
| **Evolution Understanding** | .989 | .118 | .293 | 8.373 | .000 | .757 | 1.221 |
| **Religiosity** | .056 | .024 | .080 | 2.308 | .021 | .008 | .104 |
| Biology Major | .037 | .040 | .033 | .940 | .347 | -.041 | .115 |
| Woman | .015 | .037 | .014 | .398 | .691 | -.058 | .088 |
| Born in the U.S. | -.116 | .090 | -.044 | -1.290 | .198 | -.292 | .061 |

**CATHOLIC STUDENT ONLY REGRESSIONS**

Table S19: Summary of binary logistic regression results among Catholic students only using acceptance of the common ancestry of life on Earth as the dependent variable, *Cox & Snell R^2^* = .11, (χ^2^ (5) = 209.490, p < .001)

|  |  | |  |  | |  | **95% Confidence Intervals for B** | |
| --- | --- | --- | --- | --- | --- | --- | --- | --- |
|  | **B** | **Std. Error** | **Wald** | **df** | **Significance**  **(p-value)** | **Exp(B)** | **Lower Bound** | **Upper Bound** |
| (Constant) | 1.336 | .345 | 15.003 | 1 | .000 | 3.805 |  |  |
| **Evolution Understanding** | 2.500 | .314 | 63.475 | 1 | .000 | 12.184 | 6.587 | 22.538 |
| **Religiosity** | -.710 | .066 | 115.807 | 1 | .000 | .491 | .432 | .559 |
| Biology Major | -.080 | .099 | .652 | 1 | .419 | .923 | .759 | 1.121 |
| **Woman** | -.549 | .109 | 25.569 | 1 | .000 | .578 | .467 | .715 |
| Born in the U.S. | .141 | .163 | .747 | 1 | .387 | 1.151 | .836 | 1.585 |

Table S20: Summary of linear regression results among Catholic students only using human evolution acceptance as the dependent variable, *R*^2^ = .10, *F* (5, 1871) = 40.982, *p* < .001. Variables that are significant at the .05 level are bolded. The reference group for gender is “Man” and race/ethnicity “White.”

|  | **Unstandardized Coefficients** | | **Standardized Coefficients** |  | | **95% Confidence Intervals for B** | |
| --- | --- | --- | --- | --- | --- | --- | --- |
|  | **B** | **Std. Error** | **Beta (β)** | **t** | **Significance**  **(p-value)** | **Lower Bound** | **Upper Bound** |
| (Constant) | 3.779 | .099 |  | 38.046 | .000 | 3.584 | 3.973 |
| **Evolution Understanding** | .967 | .088 | .242 | 10.973 | .000 | .795 | 1.140 |
| **Religiosity** | -.164 | .018 | -.200 | -9.107 | .000 | -.200 | -.129 |
| Biology Major | .006 | .029 | .004 | .203 | .839 | -.051 | .063 |
| **Woman** | -.077 | .031 | -.054 | -2.468 | .014 | -.138 | -.016 |
| Born in the U.S. | -.024 | .047 | -.011 | -.511 | .609 | -.117 | .069 |

Table S21: Summary of linear regression results among Catholic students only using macroevolution acceptance as the dependent variable, *R*^2^ = .08, *F* (5, 1871) = 31.955, *p* < .001. Variables that are significant at the .05 level are bolded. The reference group for gender is “Man” and race/ethnicity “White.”

|  | **Unstandardized Coefficients** | | **Standardized Coefficients** |  | | **95% Confidence Intervals for B** | |
| --- | --- | --- | --- | --- | --- | --- | --- |
|  | **B** | **Std. Error** | **Beta (β)** | **t** | **Significance**  **(p-value)** | **Lower Bound** | **Upper Bound** |
| (Constant) | 3.616 | .082 |  | 44.301 | .000 | 3.456 | 3.776 |
| **Evolution Understanding** | .795 | .072 | .244 | 10.968 | .000 | .653 | .937 |
| **Religiosity** | -.082 | .015 | -.123 | -5.531 | .000 | -.111 | -.053 |
| Biology Major | .038 | .024 | .036 | 1.605 | .109 | -.008 | .085 |
| **Woman** | -.065 | .026 | -.056 | -2.533 | .011 | -.115 | -.015 |
| Born in the U.S. | -.004 | .039 | -.002 | -.106 | .916 | -.080 | .072 |

Table S22: Summary of linear regression results among Catholic students only using microevolution acceptance as the dependent variable, *R*^2^ = .09, *F* (5, 1871) = 37.177, *p* < .001. Variables that are significant at the .05 level are bolded. The reference group for gender is “Man” and race/ethnicity “White.”

|  | **Unstandardized Coefficients** | | **Standardized Coefficients** |  | | **95% Confidence Intervals for B** | |
| --- | --- | --- | --- | --- | --- | --- | --- |
|  | **B** | **Std. Error** | **Beta (β)** | **t** | **Significance**  **(p-value)** | **Lower Bound** | **Upper Bound** |
| (Constant) | 3.617 | .080 |  | 44.973 | .000 | 3.460 | 3.775 |
| **Evolution Understanding** | .953 | .071 | .296 | 13.350 | .000 | .813 | 1.093 |
| Religiosity | -.007 | .015 | -.011 | -.513 | .608 | -.036 | .021 |
| Biology Major | .033 | .023 | .031 | 1.407 | .160 | -.013 | .079 |
| Woman | .028 | .025 | .024 | 1.096 | .273 | -.022 | .077 |
| Born in the U.S. | -.026 | .038 | -.015 | -.682 | .495 | -.101 | .049 |

**JEWISH STUDENT ONLY REGRESSIONS**

Table S23: Summary of binary logistic regression results among Jewish students only using acceptance of the common ancestry of life on Earth as the dependent variable, *Cox & Snell R^2^* = .13, (χ^2^ (5) = 20.625, p < .001)

|  |  | |  |  | |  | **95% Confidence Intervals for B** | |
| --- | --- | --- | --- | --- | --- | --- | --- | --- |
|  | **B** | **Std. Error** | **Wald** | **df** | **Significance**  **(p-value)** | **Exp(B)** | **Lower Bound** | **Upper Bound** |
| (Constant) | 4.122 | 1.734 | 5.650 | 1 | .017 | 61.695 |  |  |
| Evolution Understanding | 1.222 | 1.412 | .749 | 1 | .387 | 3.393 | .213 | 53.985 |
| **Religiosity** | -1.032 | .278 | 13.792 | 1 | .000 | .356 | .207 | .614 |
| Biology Major | -.543 | .432 | 1.578 | 1 | .209 | .581 | .249 | 1.356 |
| Woman | -.960 | .513 | 3.500 | 1 | .061 | .383 | .140 | 1.047 |
| Born in the U.S. | .555 | .932 | .355 | 1 | .552 | 1.742 | .280 | 10.834 |

Table S24: Summary of linear regression results among Jewish students only using human evolution acceptance as the dependent variable, *R*^2^ = .05, *F* (5, 147) = 2.443, *p* = .037. Variables that are significant at the .05 level are bolded. The reference group for gender is “Man” and race/ethnicity “White.”

|  | **Unstandardized Coefficients** | | **Standardized Coefficients** |  | | **95% Confidence Intervals for B** | |
| --- | --- | --- | --- | --- | --- | --- | --- |
|  | **B** | **Std. Error** | **Beta (β)** | **t** | **Significance**  **(p-value)** | **Lower Bound** | **Upper Bound** |
| (Constant) | 3.327 | .364 |  | 9.148 | .000 | 2.609 | 4.046 |
| **Evolution Understanding** | 1.084 | .320 | .269 | 3.383 | .001 | .451 | 1.717 |
| Religiosity | -.017 | .055 | -.025 | -.309 | .758 | -.126 | .092 |
| Biology Major | .061 | .094 | .052 | .647 | .519 | -.125 | .247 |
| Woman | .029 | .102 | .023 | .282 | .779 | -.172 | .230 |
| Born in the U.S. | -.019 | .207 | -.007 | -.092 | .927 | -.428 | .390 |

Table S25: Summary of linear regression results among Jewish students only using macroevolution acceptance as the dependent variable, *R*^2^ = .05, *F* (5, 147) = 2.65, *p* = .025. Variables that are significant at the .05 level are bolded. The reference group for gender is “Man” and race/ethnicity “White.”

|  | **Unstandardized Coefficients** | | **Standardized Coefficients** |  | | **95% Confidence Intervals for B** | |
| --- | --- | --- | --- | --- | --- | --- | --- |
|  | **B** | **Std. Error** | **Beta (β)** | **t** | **Significance**  **(p-value)** | **Lower Bound** | **Upper Bound** |
| (Constant) | 3.373 | .312 |  | 10.825 | .000 | 2.757 | 3.988 |
| **Evolution Understanding** | .786 | .274 | .227 | 2.865 | .005 | .244 | 1.329 |
| Religiosity | .023 | .047 | .039 | .489 | .625 | -.070 | .117 |
| Biology Major | .154 | .081 | .153 | 1.913 | .058 | -.005 | .313 |
| Woman | -.063 | .087 | -.058 | -.718 | .474 | -.235 | .110 |
| Born in the U.S. | -.028 | .177 | -.013 | -.160 | .873 | -.378 | .322 |

Table S26: Summary of linear regression results among Jewish students only using microevolution acceptance as the dependent variable, *R*^2^ = .04, *F* (5, 147) = 5.80, *p* = .061. Variables that are significant at the .05 level are bolded. The reference group for gender is “Man” and race/ethnicity “White.”

|  | **Unstandardized Coefficients** | | **Standardized Coefficients** |  | | **95% Confidence Intervals for B** | |
| --- | --- | --- | --- | --- | --- | --- | --- |
|  | **B** | **Std. Error** | **Beta (β)** | **t** | **Significance**  **(p-value)** | **Lower Bound** | **Upper Bound** |
| (Constant) | 3.884 | .332 |  | 11.701 | .000 | 3.228 | 4.540 |
| **Evolution Understanding** | .697 | .292 | .190 | 2.385 | .018 | .119 | 1.275 |
| Religiosity | -.059 | .051 | -.094 | -1.172 | .243 | -.159 | .041 |
| Biology Major | .122 | .086 | .115 | 1.424 | .156 | -.047 | .292 |
| Woman | .113 | .093 | .099 | 1.219 | .225 | -.070 | .297 |
| Born in the U.S. | .014 | .189 | .006 | .072 | .943 | -.359 | .387 |

**HINDU STUDENT ONLY REGRESSIONS**

Table S27: Summary of binary logistic regression results among Hindu students only using acceptance of the common ancestry of life on Earth as the dependent variable, *Cox & Snell R^2^* = .08, (χ^2^ (5) = 14.440, p = .013)

|  |  | |  |  | |  | **95% Confidence Intervals for B** | |
| --- | --- | --- | --- | --- | --- | --- | --- | --- |
|  | **B** | **Std. Error** | **Wald** | **df** | **Significance**  **(p-value)** | **Exp(B)** | **Lower Bound** | **Upper Bound** |
| (Constant) | 2.599 | 1.287 | 4.079 | 1 | .043 | 13.454 |  |  |
| **Evolution Understanding** | 2.579 | 1.163 | 4.921 | 1 | .027 | 13.184 | 1.350 | 128.716 |
| **Religiosity** | -.551 | .261 | 4.438 | 1 | .035 | .576 | .345 | .962 |
| Biology Major | -.124 | .453 | .075 | 1 | .784 | .883 | .363 | 2.148 |
| Woman | -.786 | .458 | 2.948 | 1 | .086 | .456 | .186 | 1.118 |
| Born in the U.S. | -.597 | .459 | 1.689 | 1 | .194 | .551 | .224 | 1.354 |

Table S28: Summary of linear regression results among Hindu students only using human evolution acceptance as the dependent variable, *R*^2^ = .12, *F* (5, 159) = 5.270, *p* < .001. Variables that are significant at the .05 level are bolded. The reference group for gender is “Man” and race/ethnicity “White.”

|  | **Unstandardized Coefficients** | | **Standardized Coefficients** |  | | **95% Confidence Intervals for B** | |
| --- | --- | --- | --- | --- | --- | --- | --- |
|  | **B** | **Std. Error** | **Beta (β)** | **t** | **Significance**  **(p-value)** | **Lower Bound** | **Upper Bound** |
| (Constant) | 2.899 | .254 |  | 11.396 | .000 | 2.396 | 3.401 |
| **Evolution Understanding** | 1.122 | .248 | .347 | 4.531 | .000 | .633 | 1.612 |
| Religiosity | .070 | .051 | .101 | 1.353 | .178 | -.032 | .171 |
| Biology Major | .094 | .092 | .077 | 1.020 | .309 | -.088 | .275 |
| Woman | -.101 | .087 | -.088 | -1.157 | .249 | -.274 | .072 |
| Born in the U.S. | .047 | .088 | .040 | .534 | .594 | -.127 | .222 |

Table S29: Summary of linear regression results among Hindu students only using macroevolution acceptance as the dependent variable, *R*^2^ = .10, *F* (5, 159) = 4.63, *p* = .001. Variables that are significant at the .05 level are bolded. The reference group for gender is “Man” and race/ethnicity “White.”

|  | **Unstandardized Coefficients** | | **Standardized Coefficients** |  | | **95% Confidence Intervals for B** | |
| --- | --- | --- | --- | --- | --- | --- | --- |
|  | **B** | **Std. Error** | **Beta (β)** | **t** | **Significance**  **(p-value)** | **Lower Bound** | **Upper Bound** |
| (Constant) | 3.267 | .237 |  | 13.788 | .000 | 2.799 | 3.735 |
| **Evolution Understanding** | .956 | .231 | .320 | 4.142 | .000 | .500 | 1.412 |
| Religiosity | -.019 | .048 | -.029 | -.392 | .696 | -.114 | .076 |
| Biology Major | .112 | .086 | .100 | 1.309 | .192 | -.057 | .281 |
| Woman | -.033 | .081 | -.031 | -.402 | .688 | -.194 | .128 |
| Born in the U.S. | .080 | .082 | .072 | .968 | .335 | -.083 | .242 |

Table S30: Summary of linear regression results among Hindu students only using microevolution acceptance as the dependent variable, *R*^2^ = .11, *F* (5, 159) = 5.02, *p* < .001. Variables that are significant at the .05 level are bolded. The reference group for gender is “Man” and race/ethnicity “White.”

|  | **Unstandardized Coefficients** | | **Standardized Coefficients** |  | | **95% Confidence Intervals for B** | |
| --- | --- | --- | --- | --- | --- | --- | --- |
|  | **B** | **Std. Error** | **Beta (β)** | **t** | **Significance**  **(p-value)** | **Lower Bound** | **Upper Bound** |
| (Constant) | 3.417 | .254 |  | 13.455 | .000 | 2.916 | 3.919 |
| **Evolution Understanding** | .977 | .247 | .303 | 3.947 | .000 | .488 | 1.465 |
| Religiosity | .001 | .051 | .001 | .010 | .992 | -.101 | .102 |
| Biology Major | .163 | .092 | .135 | 1.776 | .078 | -.018 | .344 |
| Woman | .053 | .087 | .046 | .604 | .547 | -.120 | .225 |
| Born in the U.S. | .023 | .088 | .019 | .257 | .798 | -.152 | .197 |

**BUDDHIST STUDENT ONLY REGRESSIONS**

Table S31: Summary of binary logistic regression results among Buddhist students only using acceptance of the common ancestry of life on Earth as the dependent variable, *Cox & Snell R^2^* = .10, (χ^2^ (5) = 17.368, p = .004)

|  |  | |  |  | |  | **95% Confidence Intervals for B** | |
| --- | --- | --- | --- | --- | --- | --- | --- | --- |
|  | **B** | **Std. Error** | **Wald** | **df** | **Significance**  **(p-value)** | **Exp(B)** | **Lower Bound** | **Upper Bound** |
| (Constant) | 2.933 | 1.657 | 3.131 | 1 | .077 | 18.776 |  |  |
| **Evolution Understanding** | 4.402 | 1.673 | 6.923 | 1 | .009 | 81.592 | 3.074 | 2165.965 |
| Religiosity | -.387 | .322 | 1.448 | 1 | .229 | .679 | .361 | 1.276 |
| Biology Major | -1.103 | .578 | 3.645 | 1 | .056 | .332 | .107 | 1.030 |
| Woman | -.606 | .579 | 1.096 | 1 | .295 | .545 | .175 | 1.697 |
| **Born in the U.S.** | -1.848 | .814 | 5.154 | 1 | .023 | .158 | .032 | .777 |

Table S32: Summary of linear regression results among Buddhist students only using human evolution acceptance as the dependent variable, *R*^2^ = .05, *F* (5, 167) = 2.73, *p* = .021. Variables that are significant at the .05 level are bolded. The reference group for gender is “Man” and race/ethnicity “White.”

|  | **Unstandardized Coefficients** | | **Standardized Coefficients** |  | | **95% Confidence Intervals for B** | |
| --- | --- | --- | --- | --- | --- | --- | --- |
|  | **B** | **Std. Error** | **Beta (β)** | **t** | **Significance**  **(p-value)** | **Lower Bound** | **Upper Bound** |
| (Constant) | 3.945 | .253 |  | 15.566 | .000 | 3.445 | 4.446 |
| **Evolution Understanding** | .586 | .247 | .181 | 2.374 | .019 | .099 | 1.074 |
| Religiosity | -.094 | .053 | -.135 | -1.789 | .075 | -.198 | .010 |
| Biology Major | .039 | .082 | .035 | .469 | .640 | -.124 | .201 |
| Woman | -.155 | .086 | -.136 | -1.806 | .073 | -.324 | .014 |
| Born in the U.S. | .044 | .091 | .037 | .491 | .624 | -.134 | .223 |

Table S33: Summary of linear regression results among Buddhist students only using macroevolution acceptance as the dependent variable, *R*^2^ = .05, *F* (5, 167) = 2.94, *p* = .014. Variables that are significant at the .05 level are bolded. The reference group for gender is “Man” and race/ethnicity “White.”

|  | **Unstandardized Coefficients** | | **Standardized Coefficients** |  | | **95% Confidence Intervals for B** | |
| --- | --- | --- | --- | --- | --- | --- | --- |
|  | **B** | **Std. Error** | **Beta (β)** | **t** | **Significance**  **(p-value)** | **Lower Bound** | **Upper Bound** |
| (Constant) | 3.683 | .223 |  | 16.511 | .000 | 3.242 | 4.123 |
| **Evolution Understanding** | .608 | .217 | .212 | 2.797 | .006 | .179 | 1.036 |
| Religiosity | -.051 | .046 | -.083 | -1.110 | .269 | -.142 | .040 |
| Biology Major | .040 | .072 | .041 | .553 | .581 | -.103 | .183 |
| Woman | -.077 | .075 | -.076 | -1.021 | .309 | -.226 | .072 |
| Born in the U.S. | .105 | .080 | .099 | 1.319 | .189 | -.052 | .262 |

Table S34: Summary of linear regression results among Buddhist students only using microevolution acceptance as the dependent variable, *R*^2^ = .20, *F* (5, 167) = 9.82, *p* < .001. Variables that are significant at the .05 level are bolded. The reference group for gender is “Man” and race/ethnicity “White.”

|  | **Unstandardized Coefficients** | | **Standardized Coefficients** |  | | **95% Confidence Intervals for B** | |
| --- | --- | --- | --- | --- | --- | --- | --- |
|  | **B** | **Std. Error** | **Beta (β)** | **t** | **Significance**  **(p-value)** | **Lower Bound** | **Upper Bound** |
| (Constant) | 3.426 | .232 |  | 14.757 | .000 | 2.968 | 3.885 |
| **Evolution Understanding** | 1.392 | .226 | .428 | 6.153 | .000 | .945 | 1.838 |
| Religiosity | -.037 | .048 | -.053 | -.774 | .440 | -.132 | .058 |
| Biology Major | -.117 | .075 | -.106 | -1.547 | .124 | -.266 | .032 |
| Woman | .064 | .079 | .056 | .820 | .413 | -.091 | .220 |
| Born in the U.S. | .130 | .083 | .108 | 1.561 | .120 | -.034 | .293 |

**AGNOSTIC STUDENT ONLY REGRESSSIONS**

Table S35: Summary of binary logistic regression results among Agnostic students only using acceptance of the common ancestry of life on Earth as the dependent variable, *Cox & Snell R^2^* = .06, (χ^2^ (5) = 121.501, p < .001)

|  |  | |  |  | |  | **95% Confidence Intervals for B** | |
| --- | --- | --- | --- | --- | --- | --- | --- | --- |
|  | **B** | **Std. Error** | **Wald** | **df** | **Significance**  **(p-value)** | **Exp(B)** | **Lower Bound** | **Upper Bound** |
| (Constant) | 4.881 | .696 | 49.232 | 1 | .000 | 131.818 |  |  |
| **Evolution Understanding** | 3.367 | .591 | 32.421 | 1 | .000 | 28.995 | 9.098 | 92.401 |
| **Religiosity** | -1.401 | .171 | 66.935 | 1 | .000 | .246 | .176 | .345 |
| Biology Major | -.255 | .216 | 1.383 | 1 | .240 | .775 | .507 | 1.185 |
| Woman | -.459 | .250 | 3.356 | 1 | .067 | .632 | .387 | 1.033 |
| Born in the U.S. | -.271 | .324 | .699 | 1 | .403 | .763 | .404 | 1.440 |

Table S36: Summary of linear regression results among Agnostic students only using human evolution acceptance as the dependent variable, *R*^2^ = .14, *F* (5, 1978) = 64.784, *p* < .001. Variables that are significant at the .05 level are bolded. The reference group for gender is “Man” and race/ethnicity “White.”

|  | **Unstandardized Coefficients** | | **Standardized Coefficients** |  | | **95% Confidence Intervals for B** | |
| --- | --- | --- | --- | --- | --- | --- | --- |
|  | **B** | **Std. Error** | **Beta (β)** | **t** | **Significance**  **(p-value)** | **Lower Bound** | **Upper Bound** |
| (Constant) | 3.552 | .079 |  | 45.112 | .000 | 3.398 | 3.707 |
| **Evolution Understanding** | 1.089 | .075 | .308 | 14.541 | .000 | .943 | 1.236 |
| **Religiosity** | -.131 | .019 | -.147 | -7.018 | .000 | -.167 | -.094 |
| Biology Major | .003 | .024 | .003 | .130 | .896 | -.045 | .051 |
| **Woman** | -.086 | .026 | -.070 | -3.323 | .001 | -.137 | -.035 |
| **Born in the U.S.** | .129 | .036 | .075 | 3.589 | .000 | .058 | .199 |

Table S37: Summary of linear regression results among Agnostic students only using macroevolution acceptance as the dependent variable, *R*^2^ = .12, *F* (5, 1978) = 54.992, *p* < .001. Variables that are significant at the .05 level are bolded. The reference group for gender is “Man” and race/ethnicity “White.”

|  | **Unstandardized Coefficients** | | **Standardized Coefficients** |  | | **95% Confidence Intervals for B** | |
| --- | --- | --- | --- | --- | --- | --- | --- |
|  | **B** | **Std. Error** | **Beta (β)** | **t** | **Significance**  **(p-value)** | **Lower Bound** | **Upper Bound** |
| (Constant) | 3.635 | .073 |  | 49.758 | .000 | 3.491 | 3.778 |
| **Evolution Understanding** | .874 | .069 | .269 | 12.581 | .000 | .738 | 1.011 |
| **Religiosity** | -.116 | .017 | -.142 | -6.691 | .000 | -.150 | -.082 |
| Biology Major | .036 | .023 | .034 | 1.573 | .116 | -.009 | .080 |
| **Woman** | -.096 | .024 | -.084 | -3.979 | .000 | -.143 | -.048 |
| **Born in the U.S.** | .129 | .033 | .082 | 3.889 | .000 | .064 | .194 |

Table S38: Summary of linear regression results among Agnostic students only using microevolution acceptance as the dependent variable, *R*^2^ = .13, *F* (5, 1978) = 62.43, *p* < .001. Variables that are significant at the .05 level are bolded. The reference group for gender is “Man” and race/ethnicity “White.”

|  | **Unstandardized Coefficients** | | **Standardized Coefficients** |  | | **95% Confidence Intervals for B** | |
| --- | --- | --- | --- | --- | --- | --- | --- |
|  | **B** | **Std. Error** | **Beta (β)** | **t** | **Significance**  **(p-value)** | **Lower Bound** | **Upper Bound** |
| (Constant) | 3.739 | .066 |  | 56.651 | .000 | 3.609 | 3.868 |
| **Evolution Understanding** | .934 | .063 | .316 | 14.879 | .000 | .811 | 1.057 |
| **Religiosity** | -.064 | .016 | -.086 | -4.089 | .000 | -.095 | -.033 |
| Biology Major | .006 | .021 | .006 | .286 | .775 | -.034 | .046 |
| Woman | .009 | .022 | .009 | .416 | .677 | -.034 | .052 |
| **Born in the U.S.** | .189 | .030 | .132 | 6.306 | .000 | .130 | .248 |

**ALL STUDENT REGRESSIONS**

Table S39: Summary of binary logistic regression results among all students using acceptance of common ancestry of life as the dependent variable, *Cox & Snell R^2^* = .31, (χ^2^ (5) = 2870.444, p < .001)

|  |  | |  |  | |  | **95% Confidence Intervals for B** | |
| --- | --- | --- | --- | --- | --- | --- | --- | --- |
|  | **B** | **Std. Error** | **Wald** | **df** | **Significance**  **(p-value)** | **Exp(B)** | **Lower Bound** | **Upper Bound** |
| (Constant) | 2.356 | .969 | 5.913 | 1 | .015 | 50.184 |  |  |
| Evolution Understanding | .954 | .938 | 1.033 | 1 | .310 | 9.095 | .412 | 16.327 |
| **Religiosity** | -.888 | .220 | 16.334 | 1 | .000 | .278 | .268 | .633 |
| Biology Major | .128 | .313 | .167 | 1 | .683 | 1.010 | .616 | 2.097 |
| **Woman** | -.653 | .301 | 4.703 | 1 | .030 | .685 | .289 | .939 |
| Born in the U.S. | .188 | .339 | .308 | 1 | .579 | .765 | .621 | 2.347 |

Table S40: Summary of linear regression results using among all students using human evolution acceptance as the dependent variable, *R*^2^ = .27, *F* (5, 7903) = 584.83, *p* < .001. Variables that are significant at the .05 level are bolded. The reference group for gender is “Man” and race/ethnicity “White.”

|  | **Unstandardized Coefficients** | | **Standardized Coefficients** |  | | **95% Confidence Intervals for B** | |
| --- | --- | --- | --- | --- | --- | --- | --- |
|  | **B** | **Std. Error** | **Beta (β)** | **t** | **Significance**  **(p-value)** | **Lower Bound** | **Upper Bound** |
| (Constant) | 4.053 | .048 |  | 85.053 | .000 | 3.960 | 4.147 |
| **Evolution Understanding** | .955 | .048 | .191 | 19.727 | .000 | .860 | 1.050 |
| **Religiosity** | -.318 | .007 | -.463 | -47.823 | .000 | -.331 | -.305 |
| **Biology Major** | .060 | .016 | .036 | 3.750 | .000 | .029 | .091 |
| **Woman** | -.051 | .017 | -.029 | -3.051 | .002 | -.085 | -.018 |
| Born in the U.S. | .041 | .025 | .016 | 1.633 | .102 | -.008 | .091 |

Table S41: Summary of linear regression results among all students using macroevolution acceptance as the dependent variable, *R*^2^ = .21, *F* (5, 7903) = 408.54, *p* < .001. Variables that are significant at the .05 level are bolded. The reference group for gender is “Man” and race/ethnicity “White.”

|  | **Unstandardized Coefficients** | | **Standardized Coefficients** |  | | **95% Confidence Intervals for B** | |
| --- | --- | --- | --- | --- | --- | --- | --- |
|  | **B** | **Std. Error** | **Beta (β)** | **t** | **Significance**  **(p-value)** | **Lower Bound** | **Upper Bound** |
| (Constant) | 3.928 | .040 |  | 97.703 | .000 | 3.849 | 4.006 |
| **Evolution Understanding** | .764 | .041 | .189 | 18.716 | .000 | .684 | .844 |
| **Religiosity** | -.215 | .006 | -.387 | -38.292 | .000 | -.226 | -.204 |
| **Biology Major** | .074 | .013 | .055 | 5.487 | .000 | .047 | .100 |
| **Woman** | -.045 | .014 | -.032 | -3.173 | .002 | -.073 | -.017 |
| **Born in the U.S.** | .048 | .021 | .023 | 2.267 | .023 | .007 | .090 |

Table S42: Summary of linear regression results among all students using microevolution acceptance as the dependent variable, *R*^2^ = .16, *F* (5, 7903) = 290.98, *p* < .001. Variables that are significant at the .05 level are bolded. The reference group for gender is “Man” and race/ethnicity “White.”

|  | **Unstandardized Coefficients** | | **Standardized Coefficients** |  | | **95% Confidence Intervals for B** | |
| --- | --- | --- | --- | --- | --- | --- | --- |
|  | **B** | **Std. Error** | **Beta (β)** | **t** | **Significance**  **(p-value)** | **Lower Bound** | **Upper Bound** |
| (Constant) | 3.760 | .034 |  | 109.460 | .000 | 3.692 | 3.827 |
| **Evolution Understanding** | 1.033 | .035 | .309 | 29.600 | .000 | .964 | 1.101 |
| **Religiosity** | -.097 | .005 | -.211 | -20.203 | .000 | -.106 | -.088 |
| Biology Major | .020 | .012 | .018 | 1.759 | .079 | -.002 | .043 |
| **Woman** | .032 | .012 | .027 | 2.603 | .009 | .008 | .055 |
| **Born in the U.S.** | .105 | .018 | .060 | 5.791 | .000 | .070 | .141 |

**SUMMARY OF REGRESSION COEFFICIENTS FOR COMPARISON**

Table S43: Comparison of beta coefficients across students with different religious affiliations using acceptance of the common ancestry of life on Earth as the dependent variable.

|  | Evolution Understanding | Religiosity | Biology Major | Woman | Born in US |
| --- | --- | --- | --- | --- | --- |
| Muslim | .954 | -.888*** | .128 | -.653* | .188 |
| Protestant | 1.616*** | -.832*** | .241* | -.269* | .140 |
| CJCLDS | 1.678** | -.221* | .114 | .083 | -1.001** |
| Catholic | 2.500*** | -.710*** | -.080 | -.549*** | .141 |
| Jewish | 1.222 | -1.032*** | -.543 | -.960^a^ | .555 |
| Hindu | 2.579* | -.551* | -.124 | -.786^a^ | -.597 |
| Buddhist | 4.402** | -.387 | -1.103^a^ | -.606 | -1.848* |
| Agnostic | 3.367*** | -1.401*** | -.255 | -.459^a^ | -.271 |

*p < .05, **p < .01, ***p < .001, ^a^p = .05 - .10

Table S44: Comparison of standardized Beta (β) coefficients across students with different religious affiliations using human evolution acceptance as the dependent variable.

|  | Evolution Understanding | Religiosity | Biology Major | Woman | Born in US |
| --- | --- | --- | --- | --- | --- |
| Muslim | .199** | -.423*** | .015 | -.059 | .121^a^ |
| Protestant | .167*** | -.355*** | .044* | -.015 | .024 |
| CJCLDS | .205*** | -.147*** | .070* | .013 | -.066^a^ |
| Catholic | .242*** | -.200*** | .004 | -.054* | -.011 |
| Jewish | .269*** | -.025 | .052 | .023 | -.007 |
| Hindu | .347*** | .101 | .077 | -.088 | .040 |
| Buddhist | .181* | -.135^a^ | .035 | -.136^a^ | .037 |
| Agnostic | .308*** | -.147*** | .003 | -.070*** | .075*** |

*p < .05, **p < .01, ***p < .001, ^a^p = .05 - .10

Table S45: Comparison of standardized Beta (β) coefficients across students with different religious affiliations using macroevolution acceptance as the dependent variable.

|  | Evolution Understanding | Religiosity | Biology Major | Woman | Born in US |
| --- | --- | --- | --- | --- | --- |
| Muslim | .267*** | -.223*** | -.004 | .033 | .102 |
| Protestant | .120*** | -.279*** | .080*** | .017 | .005 |
| CJCLDS | .180*** | -.048 | .053 | .009 | -.081* |
| Catholic | .244*** | -.123*** | .036 | -.056* | -.002 |
| Jewish | .227** | .039 | .153^a^ | -.058 | -.013 |
| Hindu | .320*** | -.029 | .100 | -.031 | .072 |
| Buddhist | .212** | -.083 | .041 | -.076 | .099 |
| Agnostic | .269*** | -.142*** | .034 | -.084*** | .082*** |

*p < .05, **p < .01, ***p < .001, ^a^p = .05 - .10

Table S46: Comparison of standardized Beta (β) coefficients across students with different religious affiliations using microevolution acceptance as the dependent variable.

|  | Evolution Understanding | Religiosity | Biology Major | Woman | Born in US |
| --- | --- | --- | --- | --- | --- |
| Muslim | .300*** | -.202** | -.017 | .035 | .072 |
| Protestant | .295*** | -.159*** | .032 | .059** | .059** |
| CJCLDS | .293*** | .080* | .033 | .014 | -.044 |
| Catholic | .296*** | -.011 | .031 | .024 | -.015 |
| Jewish | .190* | -.094 | .115 | .099 | .006 |
| Hindu | .303*** | .001 | .135^a^ | .046 | .019 |
| Buddhist | .428*** | -.053 | -.106 | .056 | .108 |
| Agnostic | .316*** | -.086*** | .006 | .009 | .132*** |

*p < .05, **p < .01, ***p < .001, ^a^p = .05 - .10

Additional File 3: SPSS syntax for analyses.

*SCALE RELIABILITIES*

RELIABILITY

/VARIABLES=rel1 rel2 rel3 rel4

/SCALE('ALL VARIABLES') ALL

/MODEL=ALPHA.

RELIABILITY

/VARIABLES=micro1 micro2 micro3 micro4 micro5 micro6 micro7 micro8

/SCALE('ALL VARIABLES') ALL

/MODEL=ALPHA.

RELIABILITY

/VARIABLES=human1 human2 human3 human4 human5 human6 human7 human8

/SCALE('ALL VARIABLES') ALL

/MODEL=ALPHA.

RELIABILITY

/VARIABLES=macro1 macro2 macro3 macro4 macro5 macro6 macro7 macro8

/SCALE('ALL VARIABLES') ALL

/MODEL=ALPHA.

RELIABILITY

/VARIABLES=evoint1 evoint2 evoint3 evoint4

/SCALE('ALL VARIABLES') ALL

/MODEL=ALPHA.

RELIABILITY

/VARIABLES=evound1T evound2F evound3T evound4F evound5T evound6T evound7T evound8F evound9F

evound10F evound11T evound12F evound13F evound14F

/SCALE('ALL VARIABLES') ALL

/MODEL=ALPHA.

*DESCRIPTIVES*

FREQUENCIES VARIABLES=race gender biomajor religion2

/ORDER=ANALYSIS.

*DESCRIPTIVES BY RELIGIOUS AFFILIATION*

SORT CASES BY religion2.

SPLIT FILE LAYERED BY religion2.

DESCRIPTIVES VARIABLES=evound

/STATISTICS=MEAN STDDEV MIN MAX.

DESCRIPTIVES VARIABLES=evoint

/STATISTICS=MEAN STDDEV MIN MAX.

FREQUENCIES VARIABLES=CA

/ORDER=ANALYSIS.

DESCRIPTIVES VARIABLES= human

/STATISTICS=MEAN STDDEV MIN MAX.

DESCRIPTIVES VARIABLES= macro

/STATISTICS=MEAN STDDEV MIN MAX.

DESCRIPTIVES VARIABLES= micro

/STATISTICS=MEAN STDDEV MIN MAX.

SPLIT FILE OFF.

* Encoding: UTF-8.

*REGRESSIONS FOR COMPARISONS (RQ1)*

REGRESSION

/MISSING LISTWISE

/STATISTICS COEFF OUTS CI(95) R ANOVA

/CRITERIA=PIN(.05) POUT(.10)

/NOORIGIN

/DEPENDENT evound

/METHOD=ENTER biomajor woman nonbinary asianonly blackonly latinxonly mulitracial other usborn protestant cjclds catholic jewish hindu buddhist agnostic atheist.

REGRESSION

/MISSING LISTWISE

/STATISTICS COEFF OUTS CI(95) R ANOVA

/CRITERIA=PIN(.05) POUT(.10)

/NOORIGIN

/DEPENDENT evoint

/METHOD=ENTER biomajor woman nonbinary asianonly blackonly latinxonly mulitracial other usborn protestant cjclds catholic jewish hindu buddhist agnostic atheist.

LOGISTIC REGRESSION VARIABLES CA

/METHOD=ENTER biomajor woman nonbinary asianonly blackonly latinxonly mulitracial other usborn protestant cjclds catholic jewish hindu buddhist agnostic atheist

/PRINT=CI(95)

/CRITERIA=PIN(0.05) POUT(0.10) ITERATE(20) CUT(0.5).

REGRESSION

/MISSING LISTWISE

/STATISTICS COEFF OUTS CI(95) R ANOVA

/CRITERIA=PIN(.05) POUT(.10)

/NOORIGIN

/DEPENDENT human

/METHOD=ENTER biomajor woman nonbinary asianonly blackonly latinxonly mulitracial other usborn protestant cjclds catholic jewish hindu buddhist agnostic atheist.

REGRESSION

/MISSING LISTWISE

/STATISTICS COEFF OUTS CI(95) R ANOVA

/CRITERIA=PIN(.05) POUT(.10)

/NOORIGIN

/DEPENDENT macro

/METHOD=ENTER biomajor woman nonbinary asianonly blackonly latinxonly mulitracial other usborn protestant cjclds catholic jewish hindu buddhist agnostic atheist.

REGRESSION

/MISSING LISTWISE

/STATISTICS COEFF OUTS CI(95) R ANOVA

/CRITERIA=PIN(.05) POUT(.10)

/NOORIGIN

/DEPENDENT micro

/METHOD=ENTER biomajor woman nonbinary asianonly blackonly latinxonly mulitracial other usborn protestant cjclds catholic jewish hindu buddhist agnostic atheist.

*REGRESSIONS FOR PREDICTIVE POWER OF MUSLIM STUDENTS ONLY (RQ2)*

USE ALL.

COMPUTE filter_$=(religion2 = 1).

VARIABLE LABELS filter_$ 'religion2 = 1 (FILTER)'.

VALUE LABELS filter_$ 0 'Not Selected' 1 'Selected'.

FORMATS filter_$ (f1.0).

FILTER BY filter_$.

EXECUTE.

LOGISTIC REGRESSION VARIABLES CA

/METHOD=ENTER evound religiosity biomajor woman usborn

/PRINT=CI(95)

/CRITERIA=PIN(.05) POUT(.10) ITERATE(20) CUT(.5).

REGRESSION

/MISSING LISTWISE

/STATISTICS COEFF OUTS CI(95) R ANOVA

/CRITERIA=PIN(.05) POUT(.10)

/NOORIGIN

/DEPENDENT human

/METHOD=ENTER evound religiosity biomajor woman usborn.

REGRESSION

/MISSING LISTWISE

/STATISTICS COEFF OUTS CI(95) R ANOVA

/CRITERIA=PIN(.05) POUT(.10)

/NOORIGIN

/DEPENDENT macro

/METHOD=ENTER evound religiosity biomajor woman usborn.

REGRESSION

/MISSING LISTWISE

/STATISTICS COEFF OUTS CI(95) R ANOVA

/CRITERIA=PIN(.05) POUT(.10)

/NOORIGIN

/DEPENDENT micro

/METHOD=ENTER evound religiosity biomajor woman usborn.

FILTER OFF.

USE ALL.

EXECUTE.

*REGRESSIONS FOR PREDICTIVE POWER OF JEWISH STUDENTS ONLY (RQ2)* (supplemental only)

USE ALL.

COMPUTE filter_$=(religion2 = 6).

VARIABLE LABELS filter_$ 'religion2 = 6 (FILTER)'.

VALUE LABELS filter_$ 0 'Not Selected' 1 'Selected'.

FORMATS filter_$ (f1.0).

FILTER BY filter_$.

EXECUTE.

LOGISTIC REGRESSION VARIABLES CA

/METHOD=ENTER evound religiosity biomajor woman usborn

/PRINT=CI(95)

/CRITERIA=PIN(.05) POUT(.10) ITERATE(20) CUT(.5).

REGRESSION

/MISSING LISTWISE

/STATISTICS COEFF OUTS CI(95) R ANOVA

/CRITERIA=PIN(.05) POUT(.10)

/NOORIGIN

/DEPENDENT human

/METHOD=ENTER evound religiosity biomajor woman usborn.

REGRESSION

/MISSING LISTWISE

/STATISTICS COEFF OUTS CI(95) R ANOVA

/CRITERIA=PIN(.05) POUT(.10)

/NOORIGIN

/DEPENDENT macro

/METHOD=ENTER evound religiosity biomajor woman usborn.

REGRESSION

/MISSING LISTWISE

/STATISTICS COEFF OUTS CI(95) R ANOVA

/CRITERIA=PIN(.05) POUT(.10)

/NOORIGIN

/DEPENDENT micro

/METHOD=ENTER evound religiosity biomajor woman usborn.

FILTER OFF.

USE ALL.

EXECUTE.

*REGRESSIONS FOR PREDICTIVE POWER OF HINDU STUDENTS ONLY (RQ2)* (supplemental only)

USE ALL.

COMPUTE filter_$=(religion2 = 7).

VARIABLE LABELS filter_$ 'religion2 = 7 (FILTER)'.

VALUE LABELS filter_$ 0 'Not Selected' 1 'Selected'.

FORMATS filter_$ (f1.0).

FILTER BY filter_$.

EXECUTE.

LOGISTIC REGRESSION VARIABLES CA

/METHOD=ENTER evound religiosity biomajor woman usborn

/PRINT=CI(95)

/CRITERIA=PIN(.05) POUT(.10) ITERATE(20) CUT(.5).

REGRESSION

/MISSING LISTWISE

/STATISTICS COEFF OUTS CI(95) R ANOVA

/CRITERIA=PIN(.05) POUT(.10)

/NOORIGIN

/DEPENDENT human

/METHOD=ENTER evound religiosity biomajor woman usborn.

REGRESSION

/MISSING LISTWISE

/STATISTICS COEFF OUTS CI(95) R ANOVA

/CRITERIA=PIN(.05) POUT(.10)

/NOORIGIN

/DEPENDENT macro

/METHOD=ENTER evound religiosity biomajor woman usborn.

REGRESSION

/MISSING LISTWISE

/STATISTICS COEFF OUTS CI(95) R ANOVA

/CRITERIA=PIN(.05) POUT(.10)

/NOORIGIN

/DEPENDENT micro

/METHOD=ENTER evound religiosity biomajor woman usborn.

FILTER OFF.

USE ALL.

EXECUTE.

*REGRESSIONS FOR PREDICTIVE POWER OF BUDDHIST STUDENTS ONLY (RQ2)* (supplemental only)

USE ALL.

COMPUTE filter_$=(religion2 = 8).

VARIABLE LABELS filter_$ 'religion2 = 8 (FILTER)'.

VALUE LABELS filter_$ 0 'Not Selected' 1 'Selected'.

FORMATS filter_$ (f1.0).

FILTER BY filter_$.

EXECUTE.

LOGISTIC REGRESSION VARIABLES CA

/METHOD=ENTER evound religiosity biomajor woman usborn

/PRINT=CI(95)

/CRITERIA=PIN(.05) POUT(.10) ITERATE(20) CUT(.5).

REGRESSION

/MISSING LISTWISE

/STATISTICS COEFF OUTS CI(95) R ANOVA

/CRITERIA=PIN(.05) POUT(.10)

/NOORIGIN

/DEPENDENT human

/METHOD=ENTER evound religiosity biomajor woman usborn.

REGRESSION

/MISSING LISTWISE

/STATISTICS COEFF OUTS CI(95) R ANOVA

/CRITERIA=PIN(.05) POUT(.10)

/NOORIGIN

/DEPENDENT macro

/METHOD=ENTER evound religiosity biomajor woman usborn.

REGRESSION

/MISSING LISTWISE

/STATISTICS COEFF OUTS CI(95) R ANOVA

/CRITERIA=PIN(.05) POUT(.10)

/NOORIGIN

/DEPENDENT micro

/METHOD=ENTER evound religiosity biomajor woman usborn.

FILTER OFF.

USE ALL.

EXECUTE.

*REGRESSIONS FOR PREDICTIVE POWER OF CHRISTIAN - PROTESTANT STUDENTS ONLY (RQ2)* (supplemental only)

USE ALL.

COMPUTE filter_$=(religion2 = 2).

VARIABLE LABELS filter_$ 'religion2 = 2 (FILTER)'.

VALUE LABELS filter_$ 0 'Not Selected' 1 'Selected'.

FORMATS filter_$ (f1.0).

FILTER BY filter_$.

EXECUTE.

LOGISTIC REGRESSION VARIABLES CA

/METHOD=ENTER evound religiosity biomajor woman usborn

/PRINT=CI(95)

/CRITERIA=PIN(.05) POUT(.10) ITERATE(20) CUT(.5).

REGRESSION

/MISSING LISTWISE

/STATISTICS COEFF OUTS CI(95) R ANOVA

/CRITERIA=PIN(.05) POUT(.10)

/NOORIGIN

/DEPENDENT human

/METHOD=ENTER evound religiosity biomajor woman usborn.

REGRESSION

/MISSING LISTWISE

/STATISTICS COEFF OUTS CI(95) R ANOVA

/CRITERIA=PIN(.05) POUT(.10)

/NOORIGIN

/DEPENDENT macro

/METHOD=ENTER evound religiosity biomajor woman usborn.

REGRESSION

/MISSING LISTWISE

/STATISTICS COEFF OUTS CI(95) R ANOVA

/CRITERIA=PIN(.05) POUT(.10)

/NOORIGIN

/DEPENDENT micro

/METHOD=ENTER evound religiosity biomajor woman usborn.

FILTER OFF.

USE ALL.

EXECUTE.

*REGRESSIONS FOR PREDICTIVE POWER OF CHRISTIAN - CJCLDS STUDENTS ONLY (RQ2)* (supplemental only)

USE ALL.

COMPUTE filter_$=(religion2 = 3).

VARIABLE LABELS filter_$ 'religion2 = 3 (FILTER)'.

VALUE LABELS filter_$ 0 'Not Selected' 1 'Selected'.

FORMATS filter_$ (f1.0).

FILTER BY filter_$.

EXECUTE.

LOGISTIC REGRESSION VARIABLES CA

/METHOD=ENTER evound religiosity biomajor woman usborn

/PRINT=CI(95)

/CRITERIA=PIN(.05) POUT(.10) ITERATE(20) CUT(.5).

REGRESSION

/MISSING LISTWISE

/STATISTICS COEFF OUTS CI(95) R ANOVA

/CRITERIA=PIN(.05) POUT(.10)

/NOORIGIN

/DEPENDENT human

/METHOD=ENTER evound religiosity biomajor woman usborn.

REGRESSION

/MISSING LISTWISE

/STATISTICS COEFF OUTS CI(95) R ANOVA

/CRITERIA=PIN(.05) POUT(.10)

/NOORIGIN

/DEPENDENT macro

/METHOD=ENTER evound religiosity biomajor woman usborn.

REGRESSION

/MISSING LISTWISE

/STATISTICS COEFF OUTS CI(95) R ANOVA

/CRITERIA=PIN(.05) POUT(.10)

/NOORIGIN

/DEPENDENT micro

/METHOD=ENTER evound religiosity biomajor woman usborn.

FILTER OFF.

USE ALL.

EXECUTE.

*REGRESSIONS FOR PREDICTIVE POWER OF CHRISTIAN - CATHOLIC STUDENTS ONLY (RQ2)* (supplemental only)

USE ALL.

COMPUTE filter_$=(religion2 = 5).

VARIABLE LABELS filter_$ 'religion2 = 5 (FILTER)'.

VALUE LABELS filter_$ 0 'Not Selected' 1 'Selected'.

FORMATS filter_$ (f1.0).

FILTER BY filter_$.

EXECUTE.

LOGISTIC REGRESSION VARIABLES CA

/METHOD=ENTER evound religiosity biomajor woman usborn

/PRINT=CI(95)

/CRITERIA=PIN(.05) POUT(.10) ITERATE(20) CUT(.5).

REGRESSION

/MISSING LISTWISE

/STATISTICS COEFF OUTS CI(95) R ANOVA

/CRITERIA=PIN(.05) POUT(.10)

/NOORIGIN

/DEPENDENT human

/METHOD=ENTER evound religiosity biomajor woman usborn.

REGRESSION

/MISSING LISTWISE

/STATISTICS COEFF OUTS CI(95) R ANOVA

/CRITERIA=PIN(.05) POUT(.10)

/NOORIGIN

/DEPENDENT macro

/METHOD=ENTER evound religiosity biomajor woman usborn.

REGRESSION

/MISSING LISTWISE

/STATISTICS COEFF OUTS CI(95) R ANOVA

/CRITERIA=PIN(.05) POUT(.10)

/NOORIGIN

/DEPENDENT micro

/METHOD=ENTER evound religiosity biomajor woman usborn.

FILTER OFF.

USE ALL.

EXECUTE.

*REGRESSIONS FOR PREDICTIVE POWER OF AGNOSTIC STUDENTS ONLY (RQ2)* (supplemental only)

USE ALL.

COMPUTE filter_$=(religion2 = 9).

VARIABLE LABELS filter_$ 'religion2 = 9 (FILTER)'.

VALUE LABELS filter_$ 0 'Not Selected' 1 'Selected'.

FORMATS filter_$ (f1.0).

FILTER BY filter_$.

EXECUTE.

LOGISTIC REGRESSION VARIABLES CA

/METHOD=ENTER evound religiosity biomajor woman usborn

/PRINT=CI(95)

/CRITERIA=PIN(.05) POUT(.10) ITERATE(20) CUT(.5).

REGRESSION

/MISSING LISTWISE

/STATISTICS COEFF OUTS CI(95) R ANOVA

/CRITERIA=PIN(.05) POUT(.10)

/NOORIGIN

/DEPENDENT human

/METHOD=ENTER evound religiosity biomajor woman usborn.

REGRESSION

/MISSING LISTWISE

/STATISTICS COEFF OUTS CI(95) R ANOVA

/CRITERIA=PIN(.05) POUT(.10)

/NOORIGIN

/DEPENDENT macro

/METHOD=ENTER evound religiosity biomajor woman usborn.

REGRESSION

/MISSING LISTWISE

/STATISTICS COEFF OUTS CI(95) R ANOVA

/CRITERIA=PIN(.05) POUT(.10)

/NOORIGIN

/DEPENDENT micro

/METHOD=ENTER evound religiosity biomajor woman usborn.

FILTER OFF.

USE ALL.

EXECUTE.

*analyses of RQ2 for all students (supplemental only)

LOGISTIC REGRESSION VARIABLES CA

/METHOD=ENTER evound religiosity biomajor woman usborn

/PRINT=CI(95)

/CRITERIA=PIN(.05) POUT(.10) ITERATE(20) CUT(.5).

REGRESSION

/MISSING LISTWISE

/STATISTICS COEFF OUTS CI(95) R ANOVA

/CRITERIA=PIN(.05) POUT(.10)

/NOORIGIN

/DEPENDENT human

/METHOD=ENTER evound religiosity biomajor woman usborn.

REGRESSION

/MISSING LISTWISE

/STATISTICS COEFF OUTS CI(95) R ANOVA

/CRITERIA=PIN(.05) POUT(.10)

/NOORIGIN

/DEPENDENT macro

/METHOD=ENTER evound religiosity biomajor woman usborn.

REGRESSION

/MISSING LISTWISE

/STATISTICS COEFF OUTS CI(95) R ANOVA

/CRITERIA=PIN(.05) POUT(.10)

/NOORIGIN

/DEPENDENT micro

/METHOD=ENTER evound religiosity biomajor woman usborn.
